# Lapatinib combined with neoadjuvant paclitaxel-trastuzumab-based chemotherapy in patients with human epidermal growth factor receptor 2-positive breast cancer: A meta-analysis of randomized controlled trials

**DOI:** 10.3892/ol.2015.2848

**Published:** 2015-01-05

**Authors:** JINZHONG SUN, CHUANG CHEN, XIAOLI YAO, SHENGRONG SUN

**Affiliations:** 1 Department of Breast and Thyroid Surgery, Renmin Hospital of Wuhan University, Wuhan, P.R. China; 2 Department of General Surgery, Xiangyang Hospital, Hubei University of Medicine, Xiangyang, Hubei, P.R. China

**Keywords:** lapatinib, breast cancer, neoadjuvant, meta-analysis

## Abstract

The purpose of the present study was to quantify the cumulative randomized evidence for the efficacy and safety of lapatinib combined with neoadjuvant therapy in human epidermal growth factor receptor (HER) 2-positive breast cancer. Three electronic databases, MEDLINE, Embase and Cochrane Central Register of Controlled Trials, and the abstracts of major international conferences between inception and 15 December 2013 were searched. Two evaluators independently extracted data. The end-points assessed consisted of the pathological complete response (pCR) rate, breast-conserving surgery (BCS) rate and the occurrence of adverse events. Four randomized controlled trials were assessed in the present study, involving a total of 779 participants. Compared with the patients who did not receive lapatinib, the pCR rate was higher in the hormone receptor (HR)-positive [risk ratio (RR), 1.39; 95% confidence interval (CI), 1.12–1.72; P=0.002) and HR-negative (RR, 1.38; 95% CI, 1.14–1.68; P=0.0009) patients that received lapatinib. No significant difference between the BCS rate of the two treatment arms was observed in two trials (n=382; RR, 1.14; 95% CI, 0.89–1.47; P=0.31). The primary adverse events, including diarrhea, dermatological toxicity, hepatic toxicity and neutropenia, were statistically more frequent in patients that received lapatinib (RR, 2.46; 95% CI, 1.97–3.07; P<0.00001). The present analysis revealed that the addition of lapatinib to neoadjuvant chemotherapy for HER2-positive breast cancer improves the probability of achieving a higher pCR rate, but the use of lapatinib is associated with a higher risk of adverse events.

## Introduction

Neoadjuvant chemotherapy for the treatment of breast cancer has been reported to be equivalent to adjuvant chemotherapy in terms of survival and overall disease progression (1). In addition, neoadjuvant chemotherapy offers certain attractive benefits, since it can downstage the primary tumor in the majority of patients, increasing breast-conserving surgery (BCS) rates or improving resectability (2). Neoadjuvant chemotherapy also provides an *in vivo* assessment of tumor response to chemotherapy, as the patients that attain pathological complete response (pCR) during neoadjuvant therapy exhibit a significantly improved disease-free survival rate (3,4).

Trastuzumab is a humanized monoclonal antibody that blocks the activity of human epidermal growth factor receptor (HER) 2. Combined with neoadjuvant chemotherapy for the treatment of HER2-positive breast cancer patients, trastuzumab offers a substantial benefit in terms of pCR, with no additional toxicity (5). Lapatinib is an orally-active small molecule that reversibly inhibits HER1 and HER2. As demonstrated by cell line and xenograft models (6,7), lapatinib blocks the activating signaling cascades in the MAPK and PI3K pathways, resulting in cell growth arrest or apoptosis. Clinical trials have demonstrated that lapatinib is efficacious in HER2-positive metastatic breast cancer (8).

A potential therapeutic option to improve HER2 inhibition is the combination of lapatinib and trastuzumab. The theoretical advantage of this combination is that the non-overlapping mechanism of action between the two agents and the ability to overcome the primary and acquired resistance to the agents by dual blockade. Certain randomized controlled trials (RCTs) that have explored the potential advantages by using lapatinib in the neoadjuvant setting have been previously reported (9–12).

To evaluate the efficacy and safety of lapatinib combined with neoadjuvant therapy for the treatment of HER2-positive breast cancer, a meta-analysis of all relevant published RCTs was performed.

## Patients and methods

### Eligibility criteria

The eligibility and exclusion criteria were pre-specified. Studies were considered eligible for the present meta-analysis if they were RCTs that evaluated the administration of trastuzumab-based chemotherapy compared with neoadjuvant chemotherapy using a combination of agents including lapatinib. All cytotoxic chemotherapy regimens were considered eligible for the present meta-analysis if the same chemotherapy agents were administered at the same dose in all treatment arms and that the arms differed systematically only in the anti-HER2 therapy administered. If multiple publications of the same trial or a case mix between publications was found, only the most recent or most informative publication was included. The study was approved by the ethics committee of Renmin Hospital of Wuhan University (Wuhan, China).

### Search strategy

MEDLINE (National Library of Medicine, Bethesda, MD, USA), Embase (Elsevier, Amsterdam, Netherlands) and the Cochrane Central Register of Controlled Trials (Cochrane Library, Hoboken, NJ, USA) were searched between inception and 15 December 2013, using the following searching algorithm: (neoadjuvant OR preoperative OR induction OR primary systemic OR primary chemotherapy) AND (lapatinib OR tykerb OR tyverb). The proceedings of the major international congresses, comprising the San Antonio Breast Cancer Symposium (Symposia Cancer Therapy & Research Center at UT Health Science Center San Antonio, San Antonio, TX, USA) and the American Society of Clinical Oncology Annual Meeting (American Society of Clinical Oncology, Alexandria, VA, USA), were also electronically searched to avoid the exclusion of unpublished recent trials using lapatinib in neoadjuvant chemotherapy. Finally, the reference lists of the key articles were reviewed to search for further studies.

### Data extraction

The data was independently extracted from all included studies. Disagreements were discussed to achieve consensus, or underwent third-party adjudication. From each eligible trial, the following items were recorded: authors’ name; journal name and year of publication; years of patient enrollment; country of origin; number of medical centers involved; number of patients randomized and analyzed per arm; patient age and gender; hormone receptor (HR) status; tumor size; node status; median follow-up time; technique used for HER2 identification; type and dose of chemotherapy; dose and duration of trastuzumab therapy; and dose and duration of lapatinib therapy. Primary and secondary outcome measures, consisting of pCR, BCS and all adverse events, were also recorded.

### Outcome definition

The primary outcome assessed was the rate of pCR achieved. If the primary study reported a separate pCR rate for breast tissue and breast tissue plus axilla, only the pCR rate for breast tissue plus axilla was included. The secondary outcomes assessed were the rate of BCS and all adverse events. The primary adverse events included grade 3–4 diarrhea, hepatic toxicity, dermatological toxicity and neutropenia. HR-positive was defined as immunohistochemical estrogen receptor level ≥10% or progesterone receptor level ≥10%.

### Assessment of risk of bias

Cochrane’s risk of bias tool was utilized to assess the individual risk of bias in each study (13). The criteria used for quality assessment were sequence generation of allocation, allocation concealment, masking of participants, personnel and outcome assessors, incomplete outcome data, selective outcome reporting and other sources of bias. The risk of bias in each eligible trial was independently assessed. Potential publication bias was assessed visually using a funnel plot and was statistically analyzed using Egger’s and Begg’s tests (14,15).

### Statistical analysis

Two by two tables were constructed, using the intention to treat assignment when applicable, and the risk ratio (RR) was calculated for each primary study to estimate the relative risk of each outcome in patients with HER2-positive breast cancer receiving trastuzumab-based chemotherapy versus combination of lapatinib as neoadjuvant therapy. For each eligible study group, the RR for the outcome measures was estimated and compared between the groups, and the 95 % confidence interval (CI) was also estimated. The data were then synthesized for all studies using fixed effects (Mantel-Haenszel) or random effects (Der Simonian and Laird) modeling when heterogeneity was present between studies.

The Q statistic was used to test heterogeneity between trials. The presence of statistical heterogeneity was assessed using Cochran’s Q test and quantified using I^2^ and respective 95% CIs. P<0.10 was considered to indicate a statistically significant difference. For the I^2^ values, ≥40% indicated a large heterogeneity and >75% indicated an extremely large heterogeneity. When substantial heterogeneity, classified as I^2^≥40%, was identified, subgroup analyses were performed. The subgroup analyses performed were defined *a priori* to investigate the effects of HR in pCR.

The meta-analysis was conducted using Review Manager software version 5.1 (The Cochrane Collaboration, Copenhagen, Denmark). Begg’s and Egger’s tests were performed using the Stata software package version 12.0 (StataCorp LP, College Station, TX, USA).

## Results

### Literature selection and study characteristics

**Figure 1:**
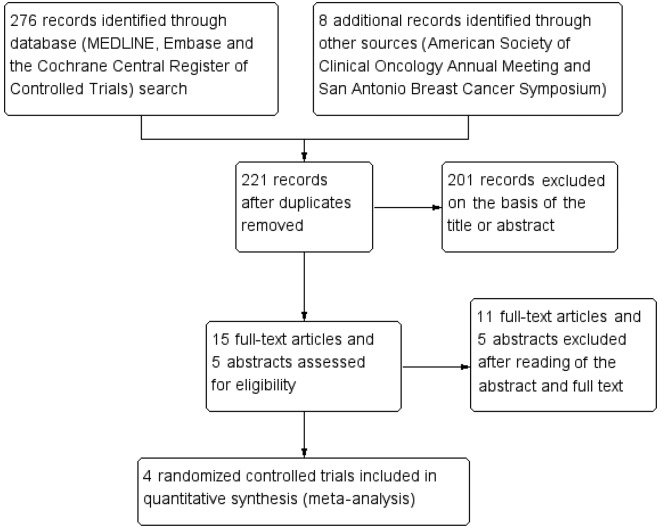
Flow chart diagram of study selection. Finally, four eligible full-text articles were retrieved.

The process of identifying eligible trials is presented in Fig. 1. The titles and abstracts of 221 unique records identified through the literature search were screened. Four eligible full-text articles were retrieved, all of which were randomized controlled trials and from peer-reviewed studies (9–12). These trials were included in the meta-analysis.

Table I reports the characteristics of the four trials that met the eligibility criteria for the present study. The four trials were three-armed and compared the administration of trastuzumab with the administration of lapatinib or a combination of the two for neoadjuvant chemotherapy, with all regimens including paclitaxel in the regimen. All trials reported the pCR rate and primary adverse events. To investigate the potential role of lapatinib in neoadjuvant therapy, only the data from the trastuzumab-based chemotherapy arm, termed the no lapatinib group, and the lapatinib plus trastuzumab-based chemotherapy arm, termed the lapatinib group, were extracted. In total, 779 patients were included in the present meta-analysis. Of those, 388 patients had been randomly allocated to the no lapatinib group and 391 to the lapatinib group.

### Risk of bias

**Figure 2:**
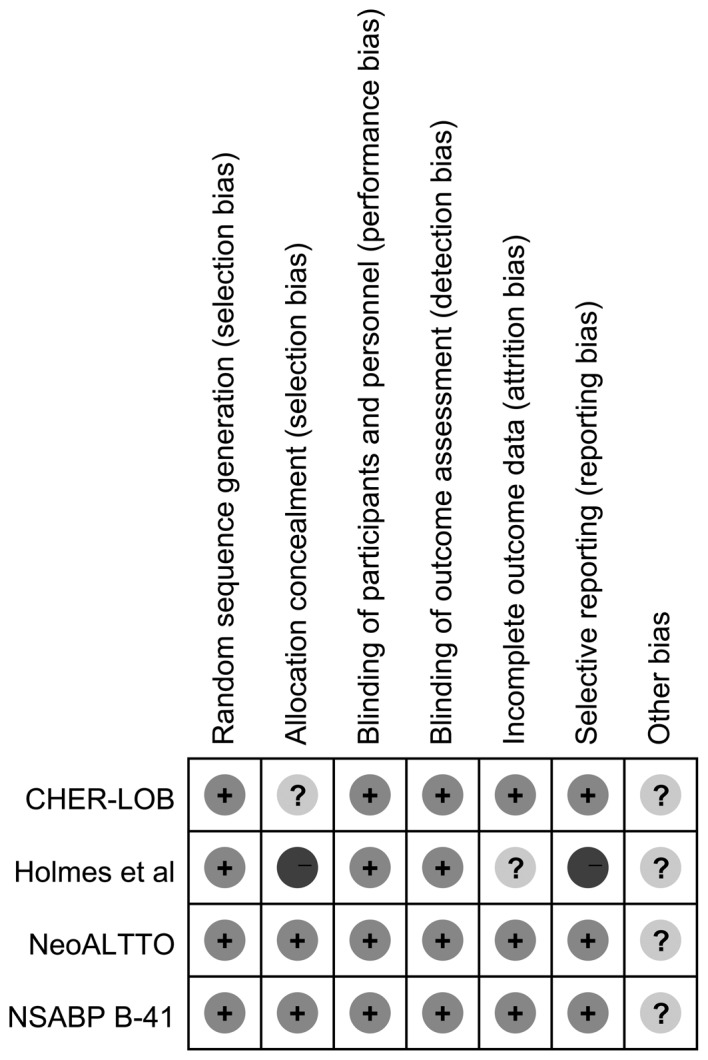
Risk of bias summary: The risk of bias was low. CHER-LOB, chemotherapy, Herceptin and lapatinib in operable breast cancer; NeoALTTO, Neo-adjuvant Lapatinib and/or Trastuzumab Treatment Organisation; NSABP, National Surgical Adjuvant Breast and Bowel Project. +, low risk; −, high risk; ?, risk unclear.

**Figure 3:**
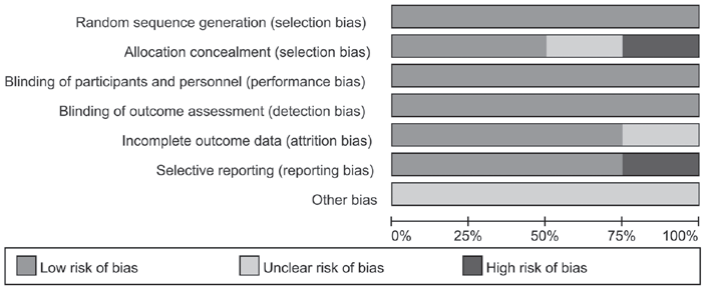
Risk of bias graph. In summary, the total risk of bias was low.

**Figure 4:**
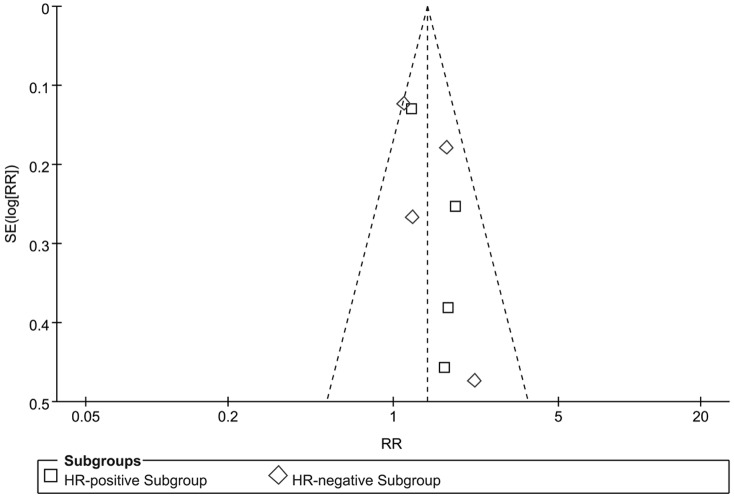
Risk of publication bias: funnel plots. A high risk of publication bias was not visually identified. HR, hormone receptor; RR, risk ratio; SE, standard error.

Two studies (9,10) were phase II clinical trials and two studies (11,12) were phase III clinical trials. The pCR rate and the primary adverse events were reported for all studies. According to the Cochrane risk of bias tool, each risk of bias item for each RCT included in the present study was assessed, and the results are summarized in Fig. 2 and presented as percentages across all included studies in Fig. 3. In summary, the total risk of bias was low. A funnel plot was drawn to assess the publication bias in terms of pCR. A high risk of publication bias was not visually identified (Fig. 4). This result was confirmed by Begg’s (P=0.216) and Egger’s (P=0.122) tests.

### Overall effect of lapatinib on pCR

**Figure 5:**
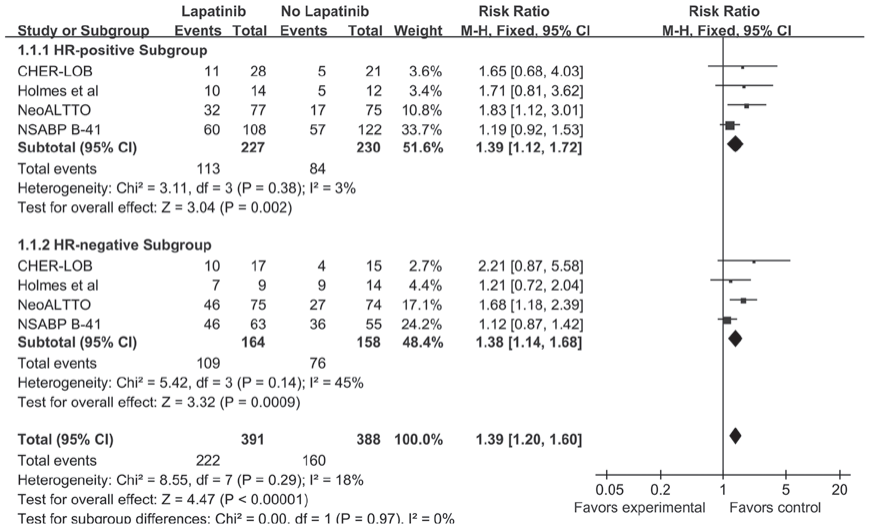
Forest plot of pathological complete response. The pathological complete response rate was significantly higher in the lapatinib group compared with the no lapatinib group for HR-positive and −negative patients. M-H, Mantel-Haenszel; CI, confidence interval; HR, hormone receptor; CHER-LOB, chemotherapy, Herceptin and lapatinib in operable breast cancer; NeoALTTO, Neo-adjuvant Lapatinib and/or Trastuzumab Treatment Organisation; NSABP, National Surgical Adjuvant Breast and Bowel Project.

All four trials, including 779 patients, included data for pCR. The absolute pCR rate was 56.78% (222 out of 391 patients) in the lapatinib group and 41.24% (160 out of 388 patients) in the no lapatinib group (RR, 1.39; 95% CI, 1.20–1.60; P<0.0001). A subgroup meta-analysis was conducted using the HR status. The probability of pCR was significantly higher in the lapatinib group compared with the no lapatinib group for HR-positive (RR, 1.39; 95% CI, 1.12–1.72; P=0.002) and HR-negative (RR, 1.38; 95% CI, 1.14–1.68; P=0.0009) patients (Fig. 5). There was no significant heterogeneity between the studies according to the subgroup analysis (HR-positive, P=0.38 and I^2^=3%; HR-negative, P=0.14 and I^2^=45%). No recurrence and survival analysis was performed due to the short-term follow-up performed in the assessed trials and a lack of the necessary data.

### Overall effect of lapatinib on the BCS rate and adverse events

Data on the number of patients that underwent BCS was available in two trials, totaling 382 patients (9,11). No difference was identified in terms of BCS between the two treatment arms (RR, 1.14; 95% CI, 0.89–1.47; P=0.31).

The primary adverse events, consisting of grade 3–4 diarrhea, dermatological toxicity, hepatic toxicity and neutropenia, were reported in all four trials. The proportion of patients that experienced primary adverse events was higher in the lapatinib group compared with the no lapatinib group (RR 2.46; 95% CI, 1.97–3.07; P<0.0001). The heterogeneity of each subgroup was moderate, with all subgroups demonstrating I^2^<40%.

**Figure 6:**
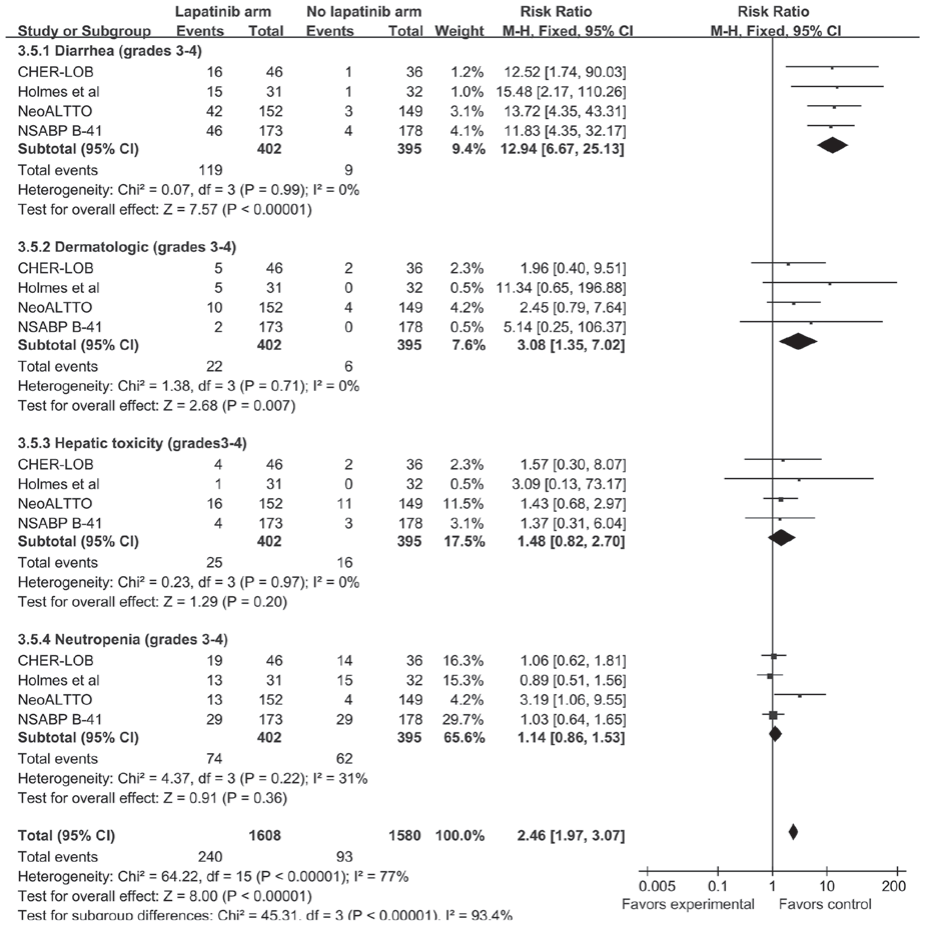
Forest plot of primary adverse events (grades 3–4). The primary adverse were statistically more frequent in patients that received lapatinib. M-H, Mantel-Haenszel; CI, confidence interval; HR, hormone receptor; CHER-LOB, chemotherapy, Herceptin and lapatinib in operable breast cancer; NeoALTTO, Neo-adjuvant Lapatinib and/or Trastuzumab Treatment Organisation; NSABP, National Surgical Adjuvant Breast and Bowel Project.

The incidence of grade 3–4 diarrhea (RR, 12.94; 95% CI, 6.67–25.13; P<0.0001) and grade 3–4 dermatological toxicity (RR, 3.08; 95% CI, 1.35–7.02; P=0.007) was significantly higher in the lapatinib group compared with the no lapatinib group. Despite there being no significant difference between the groups, grade 3–4 hepatic toxicity and neutropenia appeared to occur more frequently in the lapatinib group (Fig. 6).

The pooled RRs for additional adverse events and the 95% CI for the use of lapatinib in neoadjuvant chemotherapy versus the use of no lapatinib, are reported in Table II. No statistically significant differences were observed between the two groups, with the exception of grade 3–4 vomiting.

## Discussion

The present study, with the inclusion of all available RCTs regarding lapatinib combined to neoadjuvant therapy, provides evidence that the addition of lapatinib to neoadjuvant chemotherapy in HER2-positive breast cancer patients results in a significant increase in the pCR rate. Neoadjuvant studies using anti-HER2 agents have revealed that the pCR rate is correlated with disease-free survival (16,17). The neo-adjuvant Herceptin study (16), in which patients with HER2-positive locally advanced or inflammatory breast cancer were randomly allocated to the chemotherapy or chemotherapy plus trastuzumab groups, demonstrated a doubling in the pCR rate in the trastuzumab group compared with the chemotherapy group, and a strong correlation between the pCR rate and event-free survival was also identified. The Taxol epirubicin cyclophosphamide Herceptin neoadjuvant study reported a correlation between pCR and improved disease-free or overall survival (17). According to these previous studies, lapatinib combined with neoadjuvant therapy is likely to improve disease-free survival subsequent to additional follow-up.

The BCS rate demonstrated no statistically significant difference between the lapatinib and no lapatinib groups in the present study. However, the BCS rate is not an appropriate candidate to assess treatment effect, as breast conservation depends on several parameters, including tumor location, presence of ductal carcinoma *in situ*, breast size, contraindication to radiation therapy and patient willingness.

The use of lapatinib, as expected, was associated with two well-documented adverse events, diarrhea and dermatological toxicity (18), despite the recommended dosage reduction for lapatinib (19), the grade 3–4 diarrhea and dermatological toxicity were found to be statistically more frequent in patients receiving lapatinib in the present study. Notably, the administration of lapatinib in combination with trastuzumab did not result in an increased risk of cardiotoxicity. These results are similar to a previous meta-analysis assessing the administration neoadjuvant chemotherapy containing trastuzumab compared with no trastuzumab, in which a low cardiotoxicity was observed (5).

Anti-HER2 therapy is the treatment of choice for HER2-positive breast cancer. Dramatic clinical success has been achieved by blocking the HER-2 signaling pathway in women with breast cancer that overexpresses HER2 (20–22). The previously reported neoadjuvant study of pertuzumab and Herceptin in an early regimen evaluation trial of neoadjuvant chemotherapy investigated dual HER2 blockade using a combination of two anti-HER2 antibodies, trastuzumab and pertuzumab (23). This combination has also been found to be highly effective as a first-line treatment for HER2-positive metastatic breast cancer (24). Similar to pertuzumab, lapatinib demonstrates a complementary mechanism of action with trastuzumab, and the two exhibit a synergistic effect in preclinical models (25). In addition, a more complete HER2 blockade appears to overcome certain mechanisms of resistance to anti-HER2 agents (26,27). The effectiveness of dual blockade with trastuzumab and lapatinib has been demonstrated in the metastatic setting (28). For HER2-positive breast cancer, a previous study also supported the indication that a more complete blockade of HER receptors is an effective strategy that requires further study (29).

A previous study by Valachis *et al* (30) compared the efficacy and safety of the addition of lapatinib with the addition of trastuzumab, or a combination of the two, to neoadjuvant chemotherapy in HER2-positive breast cancer. This study included similar objectives and results on this topic to the present study. However, Valachis *et al* only analyzed two abstracts of congresses (31,32) and two full clinical trials (9,11). Due to the lack of adequate data, subgroup analyses and risk of bias assessments were not performed. The present meta-analysis provided more accurate information and identified certain different outcomes in the adverse effects compared with the study by Valachis *et al*.

The present meta-analysis possesses certain limitations that require discussion. First, the number of studies and the number of patients included in certain assessed outcomes are relatively small, which affects the power of the meta-analysis to reveal statistically significant results. Second, three trials (9,11,12) reported a separate pCR rate between breast tissue and breast tissue plus axilla. Only the pCR rate in the breast tissue plus axilla was analyzed in the present study. However, all the available randomized studies and supplementary information (10) on the topic were systematically identified.

In conclusion, based on the available evidence, the present study revealed that the administration of lapatinib in HER2-positive breast cancer in the neoadjuvant setting improves the probability of achieving a higher pCR rate, but is also associated with an increased risk of toxicity. However, the data in the literature remains to be limited. Therefore, additional follow-ups and standard randomized trials should be conducted to elucidate the role of lapatinib in neoadjuvant therapy in patients with HER2-positive breast cancer.

## Figures and Tables

**Table I:** Characteristics of eligible trials.

Clinical trial (reference)	Total number of patients, n	HER2 status assessment	Number of patients analyzed, n	Treatment arm	Number of patients per arm, n	HR-positive tumors, n (%)	Neoadjuvant chemotherapy	Neoadjuvant anti-HER2 therapy	Duration of anti-HER2 therapy, weeks
CHER-LOB (9)	121	IHC3^+^ or FISH amplification	82	No Lap	36	21 (58)	P weekly → 4 × FEC	T 4→2 mg/kg weekly	26
				Lap	46	28 (62)	P weekly → 4 × FEC	Lap 1000 mg daily + T 2 mg/kg weekly	26
Holmes *et al* (10)	100	IHC3^+^ or FISH ratio >2.2	66	No Lap	33	15 (45)	4 × FEC → P weekly	T 4→2 mg/kg weekly	26
				Lap	33	20 (61)	4 × FEC → P weekly	Lap 750 mg daily + T 2 mg/kg weekly	26
NeoALTTO (11)	455	IHC3^+^ or FISH amplification	301	No Lap	149	75 (50)	P weekly	T 4→2 mg/kg weekly	18
				Lap	152	77 (51)	P weekly	Lap 1000 mg daily + T 2 mg/kg weekly	18
NSABP B-41 (12)	529	IHC3^+^, FISH or CISH amplification	355	No Lap	181	122 (67)	4 × AC → P weekly	T 4→2 mg/kg weekly	16
				Lap	174	108 (62)	4 × AC → P weekly	Lap 750 mg daily + T 2 mg/kg weekly	16

CHER-LOB, chemotherapy, Herceptin and lapatinib in operable breast cancer; NeoALTTO, Neo-adjuvant Lapatinib and/or Trastuzumab Treatment Organisation; NSABP, National Surgical Adjuvant Breast and Bowel Project. HER2, human epidermal growth factor receptor 2; IHC, immunohistochemistry; FISH, fluorescent *in situ* hybridization; CISH, chromogenic *in situ* hybridisation; HR, hormone receptor; P, paclitaxel; Lap, lapatinib; T, trastuzumab; FEC, fluorouracil-epirubicine-cycophosphamide; AC, adriamycin-cyclophosphamide; 4 x, four cycles; →, followed.

**Table II:** Pooled analysis of other adverse events.

Adverse event	Number of trials	Events, n/total number of patients		RR	95% CI	P-value	I^2^, %

		L arm	No L arm				
Vomiting, grades 3–4	3	17/250	6/246	2.56	1.06–6.15	0.04	0
Fatigue, grades 3–4	3	14/250	12/246	1.12	0.54–2.36	0.76	0
Sensory neuropathy, grades 3–4	3	11/250	6/246	1.82	0.71–4.67	0.21	0
Mucositis, grades 3–4	2	3/219	2/214	1.31	0.27–6.24	0.74	31
Febrile neutropenia	3	14/250	11/246	1.28	0.60–2.75	0.52	0
Dyspnoea	2	17/204	18/210	0.97	0.52–1.83	0.93	40
Nausea, grades 3–4	2	9/204	2/210	3.91	0.99–15.53	0.05	0
Dehydration	2	10/204	6/210	1.66	0.64–4.34	0.30	0
CHF	3	2/341	7/326	0.50	0.03–9.48	0.65	59
LVEF decline^a^	3	1/341	2/326	0.53	0.07–3.85	0.53	0

a LVEF <50% or decline >10% from baseline.

L, lapatinib; RR, risk ratio; CI, confidence interval; CHF, congestive heart failure; LVEF, left ventricular ejection fraction.

## References

[1] Mauri D, Pavlidis N, Ioannidis JP (2005). Neoadjuvant versus adjuvant systemic treatment in breast cancer: a meta-analysis. J Natl Cancer Inst.

[2] Chia S, Swain SM, Byrd DR, Mankoff DA (2008). Locally advanced and inflammatory breast cancer. J Clin Oncol.

[3] Fisher B, Bryant J, Wolmark N (1998). Effect of preoperative chemotherapy on the outcome of women with operable breast cancer. J Clin Oncol.

[4] Kuerer HM, Newman LA, Smith TL (1999). Clinical course of breast cancer patients with complete pathologic primary tumor and axillary lymph node response to doxorubicin-based neoadjuvant chemotherapy. J Clin Oncol.

[5] Valachis A, Mauri D, Polyzos NP (2011). Trastuzumab combined to neoadjuvant chemotherapy in patients with HER2-positive breast cancer: a systematic review and meta-analysis. Breast.

[6] Xia W, Mullin RJ, Keith BR (2002). Anti-tumor activity of GW572016: a dual tyrosine kinase inhibitor blocks EGF activation of EGFR/erbB2 and downstream Erk1/2 and AKT pathways. Oncogene.

[7] Rusnak DW, Lackey K, Affleck K (2001). The effects of the novel, reversible epidermal growth factor receptor/ErbB-2 tyrosine kinase inhibitor, GW2016, on the growth of human normal and tumor-derived cell lines in vitro and in vivo. Mol Cancer Ther.

[8] Amir E, Ocaña A, Seruga B, Freedman O, Clemons M (2010). Lapatinib and HER2 status: results of a meta-analysis of randomized phase III trials in metastatic breast cancer. Cancer Treat Rev.

[9] Guarneri V, Frassoldati A, Bottini A (2012). Preoperative chemotherapy plus trastuzumab, lapatinib, or both in human epidermal growth factor receptor 2-positive operable breast cancer: results of the randomized phase II CHER-LOB study. J Clin Oncol.

[10] Holmes FA, Espina V, Liotta LA (2013). Pathologic complete response after preoperative anti-HER2 therapy correlates with alterations in PTEN, FOXO, phosphorylated Stat5, and autophagy protein signaling. BMC Res Notes.

[11] Baselga J, Bradbury I, Eidtmann H (2012). NeoALTTO Study Team: Lapatinib with trastuzumab for HER2-positive early breast cancer (NeoALTTO): a randomised, open-label, multicentre, phase 3 trial. Lancet.

[12] Robidoux A, Tang G, Rastogi P (2013). Lapatinib as a component of neoadjuvant therapy for HER2-positive operable breast cancer (NSABP protocol B-41): an open-label, randomised phase 3 trial. Lancet Oncol.

[13] Higgins JP, Altman DG, Gøtzsche PC (2011). Cochrane Bias Methods Group; Cochrane Statistical Methods Group: The Cochrane Collaboration’s tool for assessing risk of bias in randomised trials. BMJ.

[14] Egger M, Davey Smith G, Schneider M, Minder C (1997). Bias in meta-analysis detected by a simple, graphical test. BMJ.

[15] Begg CB, Mazumdar M (1994). Operating characteristics of a rank correlation test for publication bias. Biometrics.

[16] Gianni L, Eiermann W, Semiglazov V (2010). Neoadjuvant chemotherapy with trastuzumab followed by adjuvant trastuzumab versus neoadjuvant chemotherapy alone, in patients with HER2-positive locally advanced breast cancer (the NOAH trial): a randomised controlled superiority trial with a parallel HER2-negative cohort. Lancet.

[17] Untch M, Fasching PA, Konecny GE (2011). Pathologic complete response after neoadjuvant chemotherapy plus trastuzumab predicts favorable survival in human epidermal growth factor receptor 2-overexpressing breast cancer: results from the TECHNO trial of the AGO and GBG study groups. J Clin Oncol.

[18] Moy B, Goss PE (2007). Lapatinib-associated toxicity and practical management recommendations. Oncologist.

[19] Dang C, Lin N, Moy B (2010). Dose-dense doxorubicin and cyclophosphamide followed by weekly paclitaxel with trastuzumab and lapatinib in HER2/neu-overexpressed/amplified breast cancer is not feasible because of excessive diarrhea. J Clin Oncol.

[20] Slamon DJ, Leyland-Jones B, Shak S (2001). Use of chemotherapy plus a monoclonal antibody against HER2 for metastatic breast cancer that overexpresses HER2. N Engl J Med.

[21] Romond EH, Perez EA, Bryant J (2005). Trastuzumab plus adjuvant chemotherapy for operable HER2-positive breast cancer. N Engl J Med.

[22] Piccart-Gebhart MJ, Procter M, Leyland-Jones B (2005). Trastuzumab after adjuvant chemotherapy in HER2-positive breast cancer. N Engl J Med.

[23] Gianni L, Pienkowski T, Im YH (2012). Efficacy and safety of neoadjuvant pertuzumab and trastuzumab in women with locally advanced, inflammatory, or early HER2-positive breast cancer (NeoSphere): a randomised multicentre, open-label, phase 2 trial. Lancet Oncol.

[24] Baselga J, Cortés J, Kim SB (2012). CLEOPATRA Study Group: Pertuzumab plus trastuzumab plus docetaxel for metastatic breast cancer. N Engl J Med.

[25] Konecny GE, Pegram MD, Venkatesan N (2006). Activity of the dual kinase inhibitor lapatinib (GW572016) against HER-2-overexpressing and trastuzumab-treated breast cancer cells. Cancer Res.

[26] Wang YC, Morrison G, Gillihan R (2011). Different mechanisms for resistance to trastuzumab versus lapatinib in HER2-positive breast cancers - role of estrogen receptor and HER2 reactivation. Breast Cancer Res.

[27] Katiyar S, Kufareva I, Behera R (2013). Lapatinib-binding protein kinases in the African trypanosome: identification of cellular targets for kinase-directed chemical scaffolds. PLoS One.

[28] Blackwell KL, Burstein HJ, Storniolo AM (2012). Overall survival benefit with lapatinib in combination with trastuzumab for patients with human epidermal growth factor receptor 2-positive metastatic breast cancer: final results from the EGF104900 Study. J Clin Oncol.

[29] Rimawi MF, Mayer IA, Forero A (2013). Multicenter phase II study of neoadjuvant lapatinib and trastuzumab with hormonal therapy and without chemotherapy in patients with human epidermal growth factor receptor 2-overexpressing breast cancer: TBCRC 006. J Clin Oncol.

[30] Valachis A, Nearchou A, Lind P, Mauri D (2012). Lapatinib, trastuzumab or the combination added to preoperative chemotherapy for breast cancer: a meta-analysis of randomized evidence. Breast Cancer Res Treat.

[31] Robidoux A, Tang G, Rastogi P (2013). Lapatinib as a component of neoadjuvant therapy for HER2-positive operable breast cancer (NSABP protocol B-41): an open-label, randomised phase 3 trial. Lancet Oncol.

[32] Holmes FA, Nagarwala YM, Espina VA (2011). Correlation of molecular effects and pathologic complete response to preoperative lapatinib and trastuzumab, separately and combined prior to neoadjuvant breast cancer chemotherapy. J Clin Oncol (Meeting Abstracts).

